# Energy metabolism in the intestinal crypt epithelial cells of piglets during the suckling period

**DOI:** 10.1038/s41598-018-31068-3

**Published:** 2018-08-28

**Authors:** Qiye Wang, Xia Xiong, Jianzhong Li, Qiang Tu, Huansheng Yang, Yulong Yin

**Affiliations:** 1 Hunan International Joint Laboratory of Animal Intestinal Ecology and Health, Animal Nutrition and Human Health Laboratory, School of Life Sciences, Hunan Normal University, Changsha, Hunan 410007 China; 2 Key Laboratory of Agro-ecological Processes in Subtropical Region, Hunan Provincial Engineering Research Center of Healthy Livestock, Scientific Observing and Experimental Station of Animal Nutrition and Feed Science in South-Central, Ministry of Agriculture, Institute of Subtropical Agriculture, Chinese Academy of Sciences, Changsha, Hunan 410125 China; 3 Shandong University-Helmholtz Institute of Biotechnology, State Key Laboratory of Microbial Technology, School of Life Science, Shandong University, Jinan, China

## Abstract

We tested the hypothesis that energy metabolism in the intestinal crypt epithelial cells of piglets changes during the suckling period. The experiment began with 24 piglets from 8 litters (3 piglets per litter). One piglet from each litter was randomly selected and euthanized at 7, 14, or 21 d of age, respectively. Crypt cells were isolated from the mid-jejunum and protein synthesis was analyzed using isobaric tags for relative and absolute quantification. The production of proteins related to glycolysis was mainly decreased from Days 7 to 14 before increasing up to Day 21. Synthesis of proteins involved in fatty acids, amino acids (glutamate and glutamine), and citrate cycle metabolism was generally down-regulated for samples collected on Days 14 and 21 when compared with levels on Day 7. These results indicate that energy metabolism in the intestinal crypt epithelial cells changes during the suckling period. Furthermore, this pattern of metabolism varies among glucose, fatty acids, and amino acids. Therefore, these findings may be useful in efforts to regulate the intestinal development of piglets.

## Introduction

Postnatal development of the gastrointestinal tract (GIT) in piglets (*Sus scrofa*) is a very dynamic process^1^. There is an allometric growth of GIT in piglets during the first four weeks postnatal^2^. Within that first month, piglets experience an intensive change in tissues, especially in the intestinal epithelium^1,3^, as mucosal weight, villus height, and crypt depth increase in those suckling animals^1,3,4^. The intestinal mucosal weight and structure are closely associated with the cellular population^5^, which is progressively enhanced with age as the DNA content rises from 84 to 154% during the first 3 d. Such dramatic alterations may partly result from changes in epithelial cell turnover along with increased mitosis and inhibition of apoptosis^4,6^.

The intestinal epithelial cells (IEC) undergo continual cell turnover that involves the highly coordinated processes of cellular proliferation, differentiation, and apoptosis along the crypt-villus axis (CVA)^1,7,8^. This renewal is modulated by various factors that are genetic, nutritional, and hormonal^1,9,10^. Regulation of intestinal mucosal development in the neonate is a fundamental issue in the field of mucosal biology, and clarification of cellular metabolism in those epithelial cells is key to improving our knowledge about how nutrition influences mucosal development. The intestine has a high rate of energy utilization because the portal-drained viscera (PDV) (i.e., intestines, pancreas, spleen, and stomach), which contribute less than 5% of body weight (BW), account for approximately 30% of the whole-body energy expenditure and most of the energy that is utilized by the intestine^11,12^. Therefore, understanding the changes in energy metabolism in IEC during the suckling period is very important for regulating intestinal mucosal development of the neonate. In addition to glucose and fatty acids, several amino acids, especially glutamate and glutamine, have important roles in providing energy to intestinal mucosa^11,13^. Metabolism of glucose, glutamine, and fatty acids is changed in the IEC of piglets and rodents during the suckling period^1,14,15^. The metabolism of these energy substrates (glucose, glutamine, and fatty acids) differs between villus and crypt epithelial cells in piglets^1,8,14,15^. However, the previous studies on intestinal energy metabolism of suckling piglets focused on a mixture of crypt and villus epithelial cells^1^. Therefore, although crypt cell proliferation is closely related to energy metabolism^1,8^, it is unclear whether the metabolism of those energy substrates is changed during the suckling period.

We hypothesized that energy metabolism in the intestinal crypt epithelial cells of piglets is altered during the suckling period. To test this, we isolated crypt epithelial cells from mid-jejunum and investigated the synthesis of proteins during that period. Our results provide a description of the dynamics of the proteome related to nutrient uptake in the crypt cells during the suckling period.

## Results

### Changes in intestinal crypt cell protein synthesis of suckling piglets

**Figure 1:**
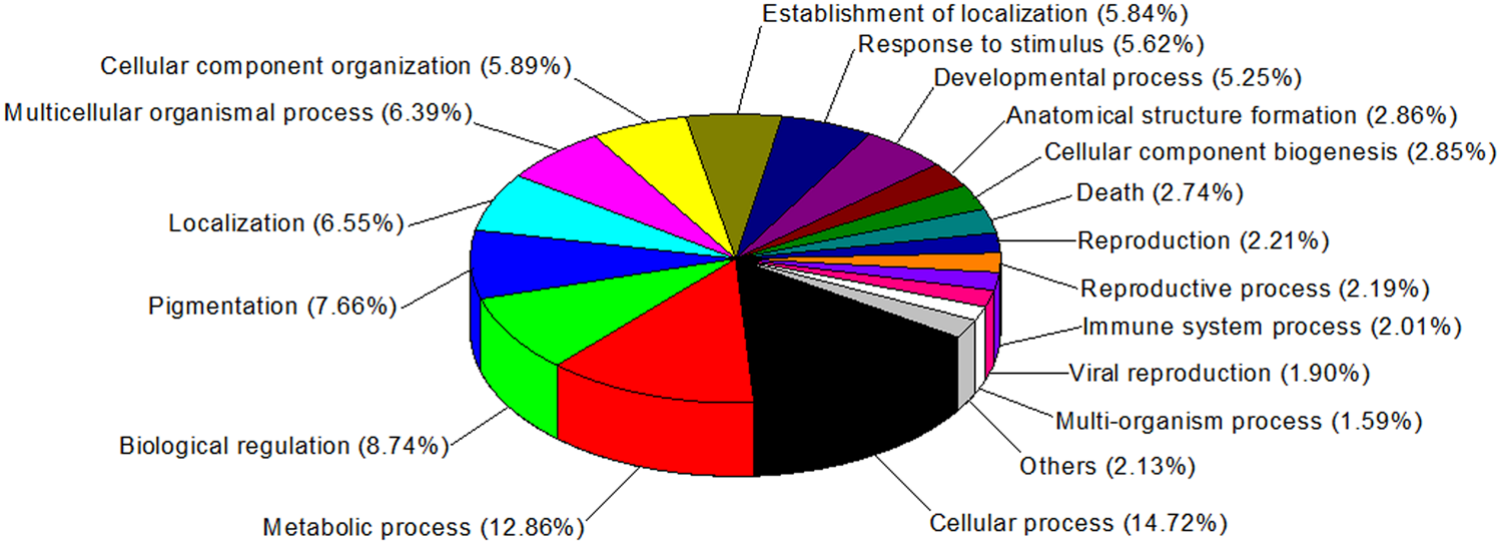
Gene Ontology terms of enrichment (based on WEGO program) for functional category of Biological Process for proteins in jejunal crypt epithelial cells of piglets during the suckling period. Any protein with ≥1.2-fold or ≤0.8-fold difference between 14 d or 21 d level and 7 d level (*P* ≤ 0.05) was considered differentially synthesized.

**Figure 2:**
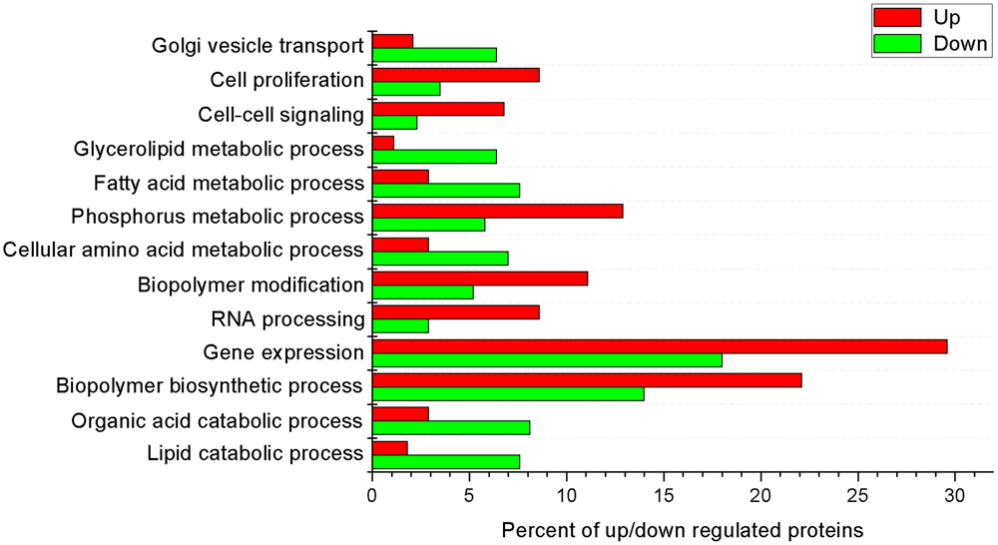
Functional categorization of proteins in jejunal crypt epithelial cells of piglets during the suckling period. Differentially synthesized proteins were grouped using Cluster 3.0 and k-means clustering, and up-regulated (red) and down-regulated (green) protein groups were selected for functional category of Biological Process using WEGO analysis. Terms having *P-*values ≤ 0.05 (Pearson Chi-Square test between numbers of Up and Down proteins) were selected.

We identified 1,004 differentially synthesized proteins in the crypt epithelial cells of mid-jejunum from 7-day-old (Average BW 2.93 kg), 14-day-old (Average BW 4.28 kg), and 21-day-old (Average BW 5.79 kg) piglets (Table S1). Biological Process Gene Ontology (GO) enrichment analysis showed that these proteins are mainly within the categories of cellular process, metabolic process, biological regulation, pigmentation, localization, multicellular organismal process, establishment of localization, response to stimulus, and developmental process (Fig. 1). They were arranged into 12 distinct groups (Clusters 1 to 12; Fig. S1) through k-means clustering. Whereas the synthesis of proteins in Cluster 5 was down-regulated during the suckling period, that of proteins in Cluster 12 was up-regulated. We then used the Web Gene Ontology (WEGO)^16^ program to analyze the GO enrichment of down-regulated (Down) proteins and up-regulated (Up) proteins during the suckling period (compared with detection at 7 d). The proteins among the Biological Process GO terms that differed significantly (*P* < 0.05) in their numbers between Down and Up regulation were also selected by using WEGO program (Fig. 2). The percentage of proteins related to lipid catabolic process, organic acid catabolic process, cellular amino acid metabolic process, fatty acid metabolic process, and Golgi vesicle transport was significantly higher in the Down group while the percentage of proteins involved in biopolymer biosynthetic process, gene expression, RNA processing, biopolymer modification, phosphorus metabolic process, cell-cell signaling, and cell proliferation was significantly higher in the Up group.

### Glycolysis and fatty acid metabolism

**Figure 3:**
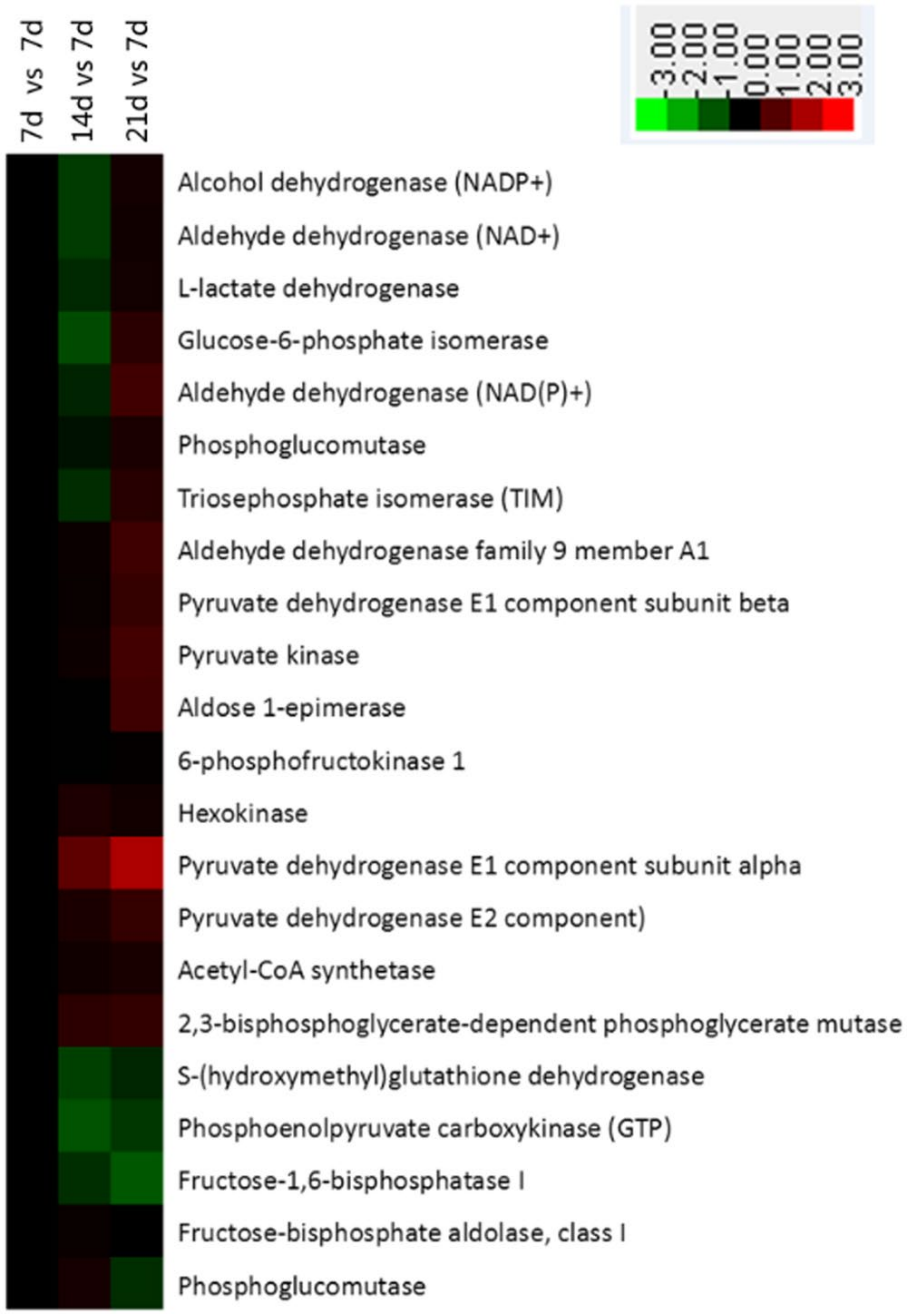
Enrichment of proteins in glycolysis pathway in jejunal crypt epithelial cells of suckling piglets based on analysis using KEGG database. Any protein with ≥1.2-fold or ≤0.8-fold difference between 14 d or 21 d level and 7 d level (*P* ≤ 0.05) was considered differentially synthesized. Up-regulated and down-regulated proteins are shown in red and green, respectively.

**Figure 4:**
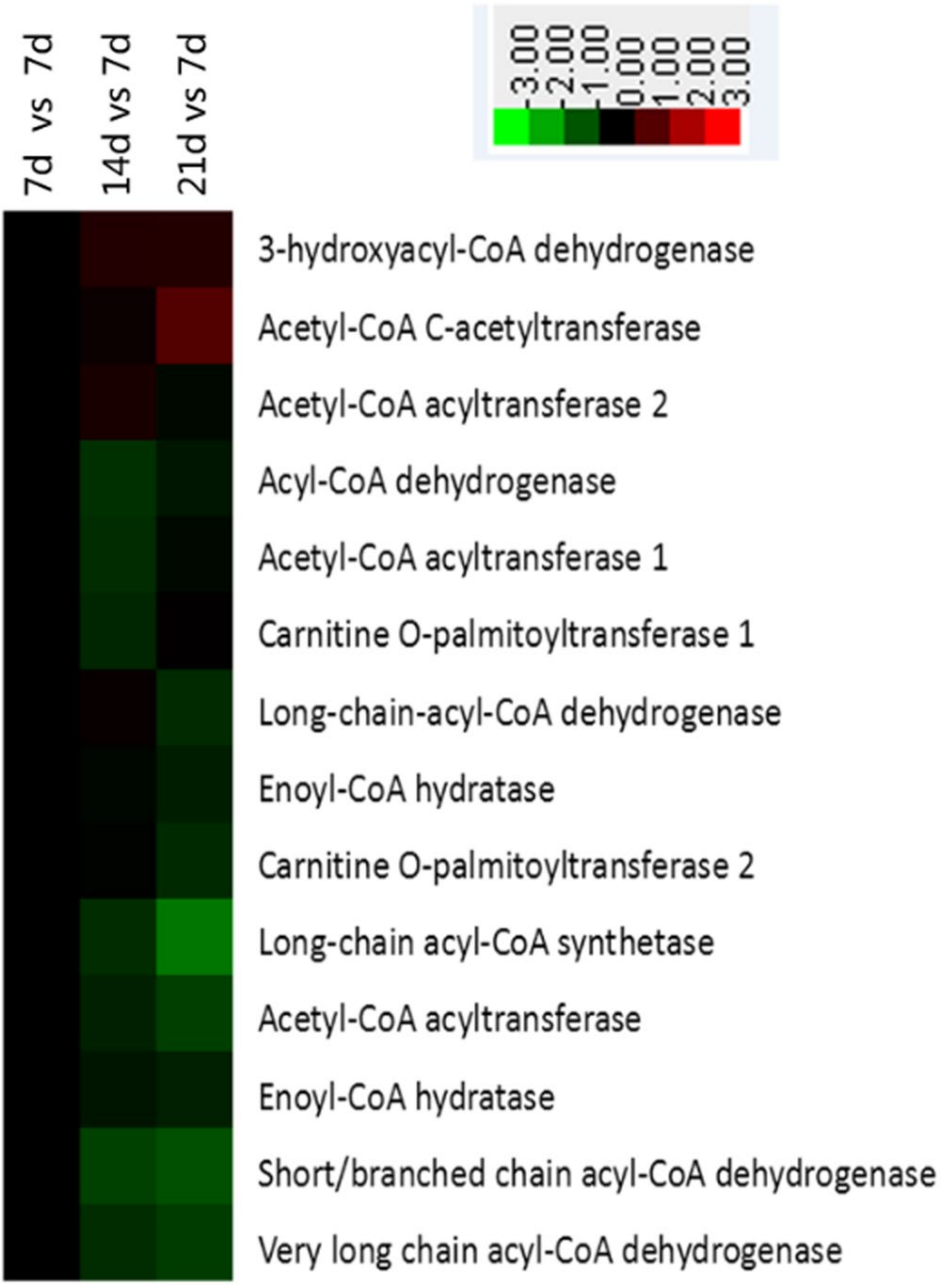
Enrichment of proteins in fatty acid metabolism pathway in jejunal crypt epithelial cells of suckling piglets based on analysis using KEGG database. Any protein with ≥1.2-fold or ≤0.8-fold difference between 14 d or 21 d level and 7 d level (*P* ≤ 0.05) was considered differentially synthesized. Up-regulated and down-regulated proteins are shown in red and green, respectively.

According to the KEGG database, 22 differentially synthesized glycolysis proteins were enriched in the intestinal crypt epithelial cells of suckling piglets (Fig. 3). Of those, nine were down-regulated and four were up-regulated in the intestines of 14-day-old animals when compared with samples taken in Day 7. On Day 21, 12 proteins were up-regulated and four were down-regulated when compared with the status on Day 7. Among the 14 that showed roles in fatty acid metabolism, the synthesis of seven (Day 14) and eight (Day 21) proteins was down-regulated in the crypt cells (Fig. 4).

### Alanine, aspartate, and glutamate metabolism and the citrate cycle

**Figure 5:**
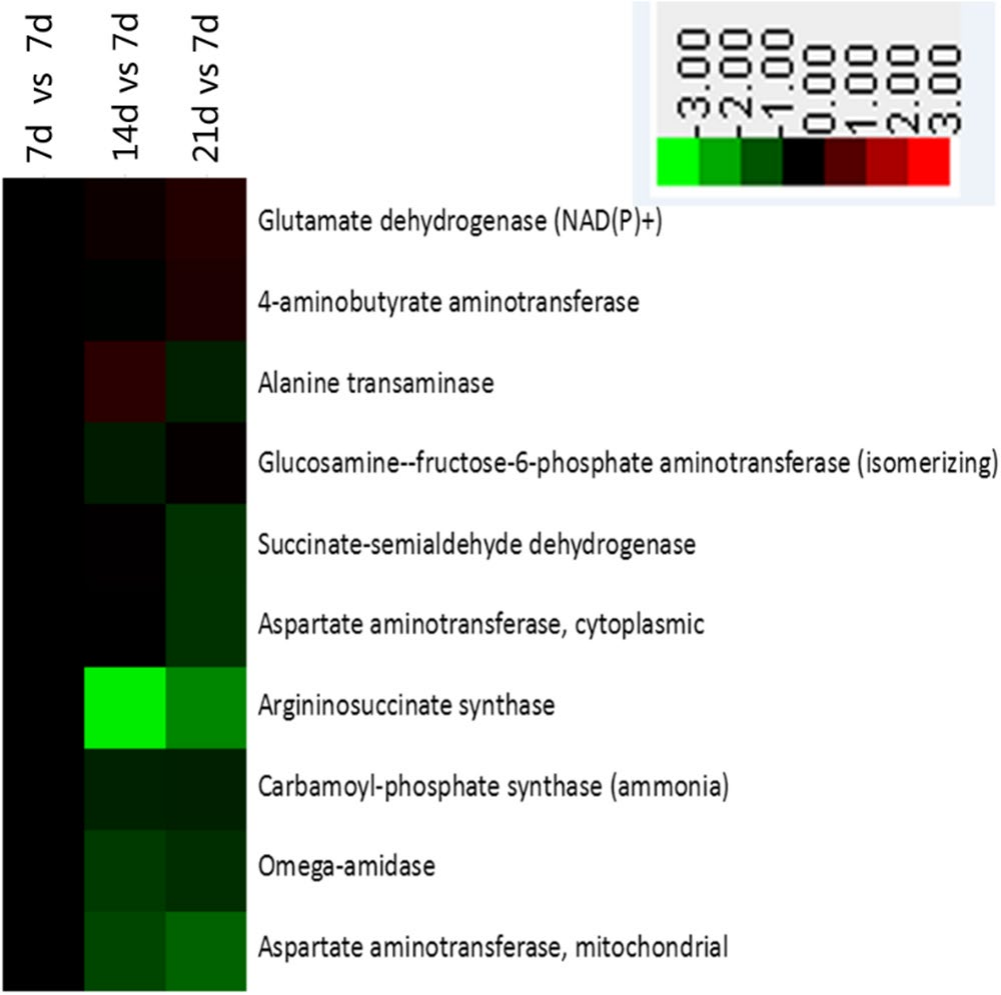
Enrichment of proteins in alanine, aspartate, and glutamate metabolism pathway in jejunal crypt epithelial cells of suckling piglets based on analysis using KEGG database. Any protein with ≥1.2-fold or ≤0.8-fold difference between 14 d or 21 d level and 7 d level (*P* ≤ 0.05) was considered differentially synthesized. Up-regulated and down-regulated proteins are shown in red and green, respectively.

**Figure 6:**
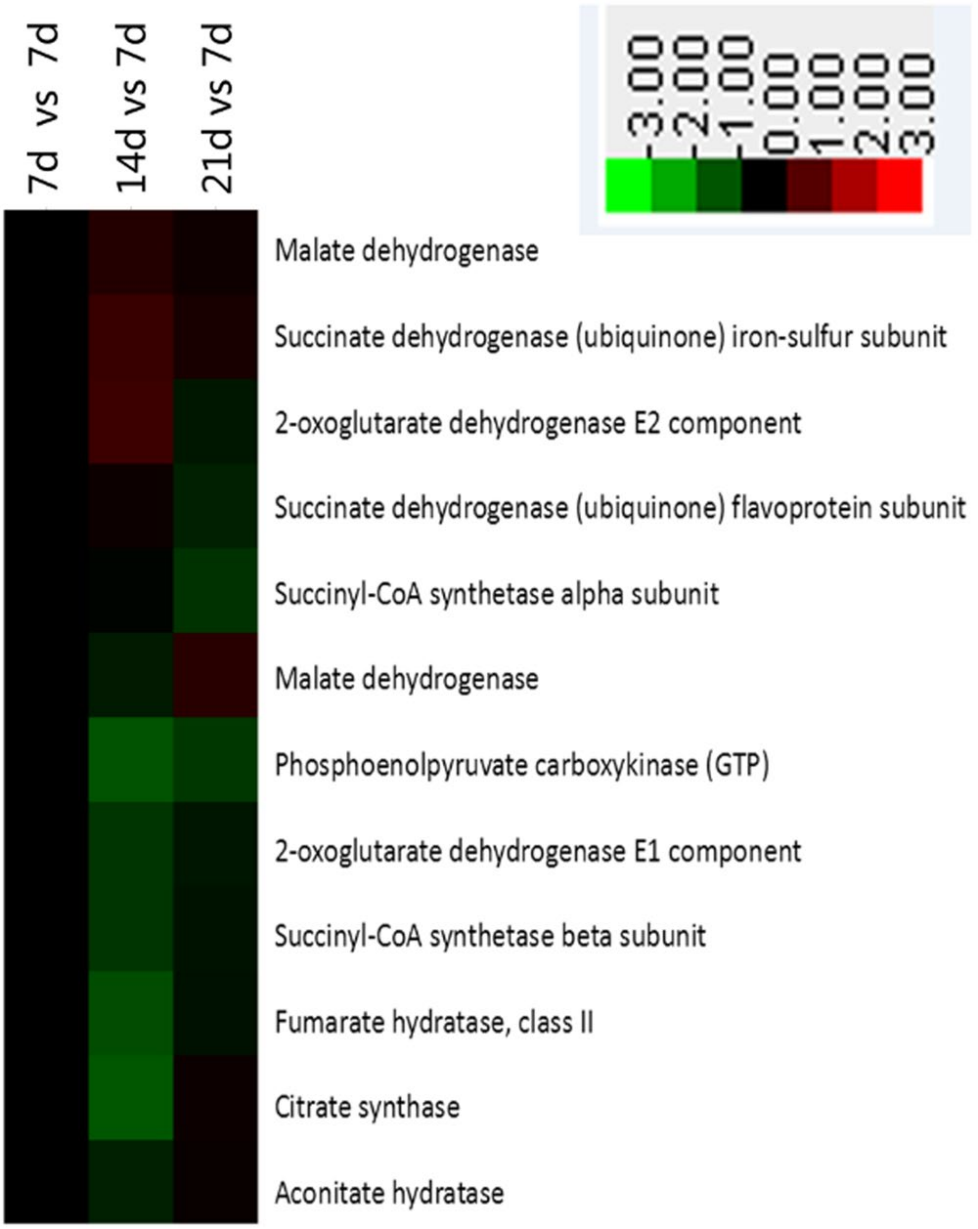
Enrichment of proteins in citrate cycle pathway in jejunal crypt epithelial cells of suckling piglets based on analysis using KEGG database. Any protein with ≥1.2-fold or ≤0.8-fold difference between 14 d or 21 d level and 7 d level (*P* ≤ 0.05) was considered differentially synthesized. Up-regulated and down-regulated proteins are shown in red and green, respectively.

We identified 10 proteins related to alanine, aspartate, and glutamate metabolism (Fig. 5). Of those, five were down-regulated in the crypt cells on Day 14 (versus Day 7) while eight were down-regulated on Day 21 (Fig. 5). Of the 12 related to the citrate cycle, six proteins were down-regulated in 14 d piglets compared with 7 d piglets, while only three were down-regulated and two were up-regulated on Day 21 when compared with the 7 d baseline (Fig. 6).

### Cell proliferation

**Figure 7:**
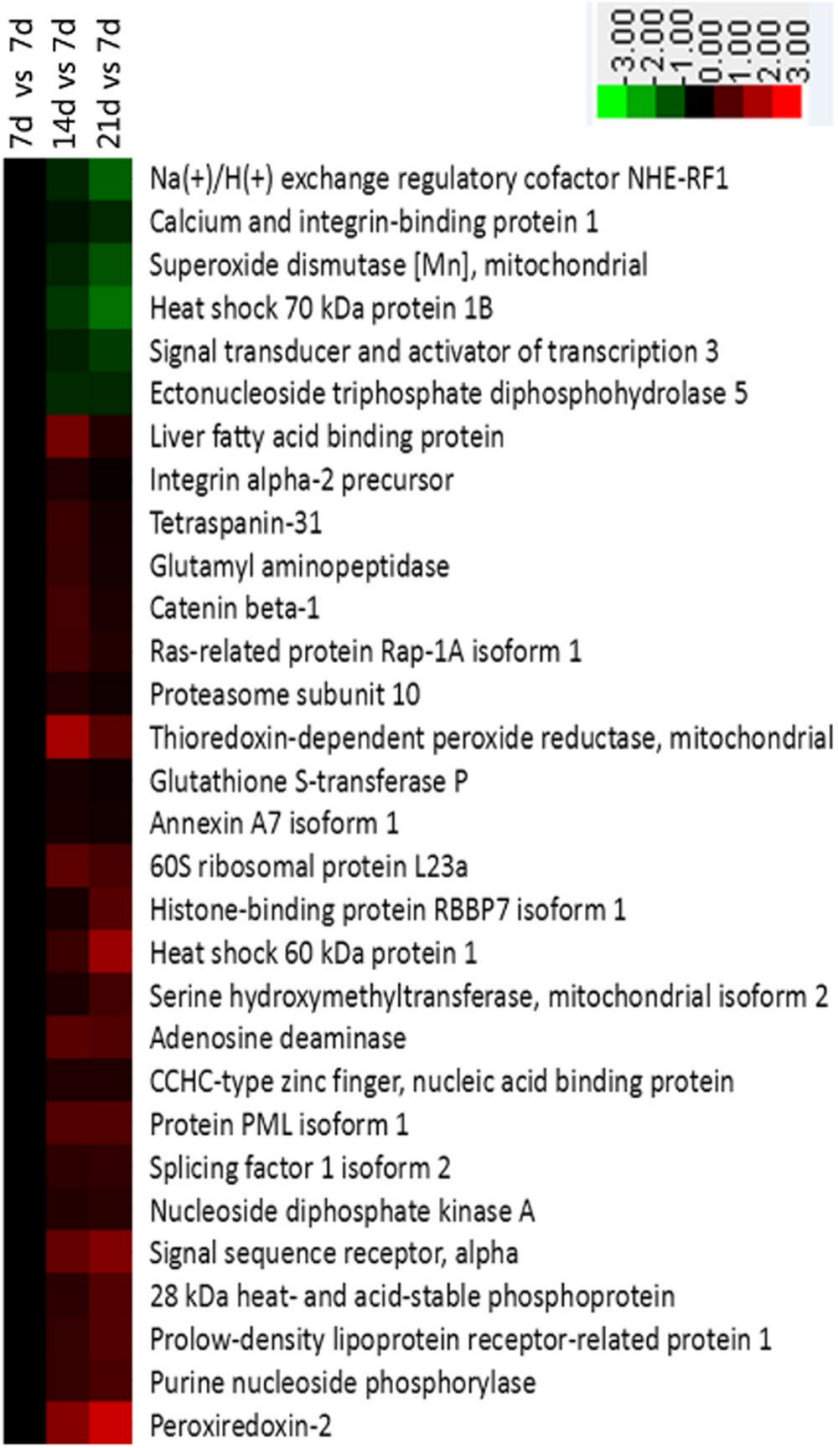
Proteins involved in cell proliferation in jejunal crypt epithelial cells of suckling piglets, as selected and grouped via Cluster 3.0. Relationships were significant (*P* ≤ 0.05) between up-regulated (red) and down-regulated (green) proteins, based on WEGO analysis.

In all, 30 differentially synthesized proteins were enriched in cell proliferation according to the WEGO program (Fig. 7). Of those, 24 and 18 were up-regulated in the intestinal crypt epithelial cells of 14 d and 21 d piglets, respectively, when compared with 7 d piglets. Fewer than six were down-regulated by Days 14 and 21 versus levels on Day 7 (Fig. 7).

## Discussion

Piglets are usually weaned between three and four weeks of age^1,17^. During this suckling period, the GIT exhibit an allometric growth in piglets during the first four weeks postnatal^1,2^. For example, the weight of the small intestine increases by more than six-fold within the first four weeks postnatal^18,19^. The most significant changes occur in intestinal mucosa^1,20^. First, the vacuolated fetal-type enterocytes in the small intestine of a neonatal piglet are gradually replaced by non-vacuolated adult-type enterocytes within 21 d after birth^20^. Second, the height, size, shape, and density of the small intestine villi are significantly altered during the first three weeks^3^ along with the rate of cell proliferation and the depth of the crypt^20^. Similar increases in the weight and size of the small intestine, the numbers of crypt and villi cells, and cell proliferation in the crypt have been reported for mice and rats^5,21^. We found here that the synthesis of proteins related to cell proliferation increased primarily in the crypt, which supports the results from previous studies demonstrating a relationship between piglet lactation and crypt cell proliferation. Further examinations are needed to determine the mechanism that directs this type of proliferation.

The small intestine is not only the primary organ responsible for nutrient digestion and absorption but is also responsible for the high rate of nutrient expenditure^11^. Moreover, the PDV tissues account for more than 50% of the whole-body turnover of some essential amino acids^22^. This organ plays key roles in nutrient utilization because the intestinal mucosa have a high rate of cell turnover, and nutrient intake by the IEC requires energy^11^. We found here that more than 12% of the differentially synthesized proteins are involved in the category of metabolic process in crypt epithelial cells from suckling piglets. This indicates that nutrient partitioning in those cells affects their proliferation because the processes associated with lipid and organic acid catabolism were generally inhibited while those related to biosynthesis (e.g., biopolymer biosynthetic process, gene expression, RNA processing, and biopolymer modification) were mainly enhanced. Cell proliferation requires the synthesis of a large number of molecules. In addition, energy metabolism in IEC is increased along the CVA^8^. Therefore, additional research is necessary to monitor changes in energy metabolism in villus epithelial cells.

Energy metabolism is more complex in piglet intestinal mucosa than in other tissues. First, its energy source is from both arterial and luminal substrates, and the pattern of substrate oxidation depends upon the composition of dietary nutrients^12,23^. Second, amino acids, especially glutamate and glutamine, rather than glucose and fatty acids, are the primary energy substrates, accounting for 19% (glutamine), 36% (enteral glutamate), 15% (enteral glucose), and 29% (arterial glucose) of the total CO_2_ production in the PDV^13,24^. Third, energy metabolism in the IEC is changed during maturation along the CVA and that of most of substrates, except glutamine, is increased there^8^. Our results showed that the synthesis of proteins involved in the metabolism of fatty acids and amino acids was mainly decreased in crypt cells during the suckling period, while the synthesis of proteins related to glucose metabolism was mainly increased. In the IEC of mice, the synthesis of proteins participating in fatty acid metabolism is greater at 7 d of age than at 14 d^25^. Glucose oxidation in piglet IEC decreases during the suckling period, which is in contrast to our results showing that the synthesis of proteins related to glycolysis was mainly increased^26^. This inconsistency between reports might be because Wu *et al*. measured the production of CO_2_ from glucose while we simply tested the synthesis of glycolysis-related proteins, and those products may not have finally led to CO_2_. Furthermore, we used only crypt cells in our current experiments while the previous work involved whole IEC. Nevertheless, both of these investigations showed that glutamine oxidation is diminished in the IEC of suckling piglets^26^. Metabolism changes in crypt epithelial cells during suckling suggest that energy is a critical factor in gastrointestinal development. Further studies are needed to determine whether oral administration of energy substrates can affect suckling pig crypt function and gastrointestinal development. Piglets often develop gastrointestinal diseases that are accompanied by villus atrophy; crypt hyperplasia; and, impaired GIT development^27,28^. These adversely affect intestinal energy metabolism resulting in decreased feed intake^27,28^. Early piglet weaning results in intestinal dysfunction as there is a transient drop in energy intake. The results of this study may assist in selecting energy substrates for weaning piglets^28^.

The metabolism of IEC can be affected by both arterial and luminal nutrients. Although we noted that the metabolism of glucose, amino acids, and fatty acids changed in the crypt epithelial cells between Days 7 and 21, other researchers have found that the contents of fat, lactose, and proteins (glutamate and glutamine) do not vary in sow milk during the suckling period^29–31^. Therefore, the alterations in energy metabolism in our suckling piglets did not depend upon luminal nutrients. It was reported that the crypt epithelial cells may be more dependent on arterial nutrients while villus epithelial cells are more depend on luminal nutrients^32^. The changes in energy metabolism of crypt epithelial cells in piglets during the suckling period may be affected by arterial nutrients. More studies will be needed to test the affects of arterial nutrients on the energy metabolism of intestinal crypt epithelial cells during the suckling period. Because the development of intestinal mucosa is affected by genetic programming during the suckling period, such metabolic changes in piglet crypt cells might also be controlled by genetic factors^1^.

In conclusion, our results demonstrate that metabolic alterations occur in intestinal crypt epithelial cells of piglets during the suckling period. While glucose metabolism increases, that of fatty acids and amino acids decreases. These findings provide support for a potential means to regulate intestinal development in suckling piglets.

## Materials and Methods

The experimental design and procedures in this study were reviewed and approved by the Animal Care and Use Committee of the Institute of Subtropical Agriculture, Chinese Academy of Science. The animal experiments were carried out in accordance with the standards of the International Guiding Principles for Biomedical Research Involving Animals.

### Animals and isolation of intestinal crypt epithelial cells

We selected 24 piglets (Duroc × [Landrace × Yorkshire]) from eight litters (3 piglets per litter) based on body weight (medium) and sex (male). The sows had free access to feed and drinking water and the piglets had free access to nipple and drinking water during the experimental period. At 7, 14, and 21 d of age, one piglet from each litter was randomly selected and maintained under general anesthesia before being sacrificed with an intravenous (jugular vein) injection of 4% sodium pentobarbital solution (40 mg/kg body weight; Sigma, St. Louis, MO, USA). Crypt epithelial cells were isolated from the middle jejunum by the distended intestinal sac method, as previously described^8,33,34^. Briefly, jejunum segments were first rinsed in ice-cold physiological saline solution and then incubated with oxygenated phosphate-buffered saline at 37 °C for 30 min. They were then incubated with oxygenated isolation buffer [5 mM Na_2_EDTA, 10 mM HEPES (pH 7.4), 0.5 mM DTT, 0.25% BSA, 2.5 mM D-glucose, 2.5 mM L-glutamine, and 0.5 mM dl-β-hydroxybutyrate sodium salt, oxygenated with an O_2_/CO_2_ mixture (19:1, v/v)] at 37 °C for 40 min to isolate the upper villus epithelial cells. The intestine segments were then incubated for 50 min to obtain the middle villus epithelial cells, followed by a 60-min incubation to isolate the crypt epithelial cells. The isolation buffers were collected and centrifuged (400 × g, 4 min, 4 °C). The collected epithelial cells were washed twice with an oxygenated cell suspension buffer [10 mM HEPES, 1.5 mM CaCl_2_, and 2.0 mM MgCl_2_ (pH 7.4)]. All of the chemicals used for cell isolation were obtained from Sigma, except for the dl-β-hydroxybutyrate (J&K Chemical Ltd., Beijing, China). The isolated cells were confirmed by comparing the activity of alkaline phosphatase (Alp) with the synthesis of proliferating cell nuclear antigen (PCNA) along the CVA because Alp activity increases there while the abundance of PCNA decreases^8^.

### Sample preparation and isobaric labeling

A lysis buffer composed of 7 M urea, 2 M thiourea, 4% w/v 3-[(3-cholamidopropyl) dimethylammonio] propanesulfonate (Sigma, St. Louis, MO, USA), 20 mM tributyl phosphate (Sigma, St. Louis, MO, USA), and 0.2% Bio-lyte (pH 3–10) plus a protease inhibitor cocktail (Roche Diagnostics Ltd, Mannheim, Germany) were used to re-suspend and disrupt the isolated cells. The lysate was then treated with DNAse I (Qiagen, Hilden, Germany) and RNAse A (Thermo Fisher Scientific, Waltham, MA, USA) at final concentrations of 1 mg/mL and 0.25 mg/mL, respectively. The protein solution was separated from the cell debris by centrifugation (12,000 × g, 4 °C, 5 min). A Ready Prep 2-D Cleanup Kit (Bio-Rad Laboratories, Hercules, CA, USA) was used for further purification of the crude protein, which was then subjected to a reductive alkylation reaction. The protein concentration was measured with a 2-D Quant Kit (GE Healthcare, San Diego, CA, USA). Afterward, trypsin digestion and iTRAQ labeling were performed according to the manufacturer’s protocol (Applied Biosystems, Foster City, CA, USA). Briefly, 100 μg of total protein per sample was reduced and alkylated, then digested overnight with trypsin (Promega, Madison, WI, USA) at 37 °C. It was labeled with iTRAQ-reagents (Applied Biosystems) as follows: 7 days, iTRAQ reagent 115; 14 days, iTRAQ reagent 116; and 21 days, iTRAQ reagent 117.

### Peptide fractionation and LC-MS/MS acquisition

The isotopically labeled samples were pooled and an Ultremex SCX column containing 5-μm particles (Phenomenex, Torrance, CA, USA) was used to obtain 12 fractions. After a Strata X C18 column (Phenomenex) was used to de-salt the eluted fractions, they were dried under vacuum. The average peptide concentration in each fraction was adjusted to 0.25 μg/μL. Dried peptides were stored at −80 °C prior to mass-spectrometer (MS) analysis. Analytical separations were performed using a nanospray ion source system (Waters, Milford, MA, USA), coupled with Triple TOF. Symmetry C18 (5 μm; 180 μm × 20 mm)-packed microfluidic traps and nanofluidic columns were employed for online trapping and de-salting, while BEH130 C18 (1.7 μm; 100 μm × 100 mm)-packed nanofluidic columns were used for the separations. The mobile phase included water/acetonitrile/formic acid solvents (A: 98/2/0.1% and B: 2/98/0.1%). One portion of a 2.25 μg (9 μL) sample was loaded, trapped, and de-salted, and an analytical separation was established by maintaining 5% B for 1 min at a flow rate of 300 nL/min. In the following 64 min, a linear gradient to 35% B occurred over 40 min. The level was increased to 80% B over 5 min and maintained for 5 min after the peptide elution window. Initial chromatographic conditions were restored in 2 min.

A Triple TOF 5600 System (AB SCIEX, Framingham, MA, USA), fitted with a Nanospray III source (AB SCIEX), was used with a pulled quartz tip as the emitter (New Objectives, Woburn, MA, USA) for data acquisition. Conditions included an ion spray voltage of 2.5 kV, curtain gas of 30 Psi, nebulizer gas of 15 Psi, and an interface heater temperature of 150 °C. For the TOF MS scans, the MS was operated with a RP ≥ 30,000 FWHM. For information dependent acquisition (IDA), survey scans were acquired in 250 ms, and as many as 30 product ion scans were collected if they exceeded a threshold of 120 counts per second with a 2+ to 5+ charge-state. The total cycle time was fixed to 3.3 s and the Q2 transmission window was 100 Da for 100%.

Four time bins were summed for each scan at a pulser frequency value of 11 kHz by monitoring the 40 GHz multichannel TDC detector with four-anode/channel detection. A sweeping collision energy setting of 35 ± 5 eV, coupled with iTRAQ adjust rolling collision energy, was applied to all precursor ions for collision-induced dissociation. The dynamic exclusion was set at 1/2 of the peak width (18 s), and the precursor was then refreshed off the exclusion list.

### Database analysis and quantification

Mascot software (version 2.3.02; Matrix Science, Boston, MA, USA) was used to identify and quantify proteins. Searches were made against the NCBI non-redundant database that consisted of *Sus scrofa* proteins. Spectra from the 12 fractions of each sample were combined into one Mascot generic format (MGF) file after the raw data were loaded, and the MGF file was searched based on the following parameters: (i) trypsin was selected as the enzyme, with one missed cleavage allowed; (ii) the fixed modifications of carbamidomethylation were set as Cys; and (iii) peptide tolerance was set at 0.05 Da, and MS/MS tolerance was set at 0.1 Da. An automatic decoy database search strategy was used to estimate the false discovery rate (FDR), which was calculated as the number of false-positive matches divided by the total number of matches. The final FDR was <1.5%. All search results were passed through additional filters before data exportation. For protein identification, the filters included a significance threshold *P* ≤ 0.05 (95% confidence) and an ion score or expected cutoff <0.05 (95% confidence). For protein quantitation, the filters included “median”, used for the protein ratio type; a minimum precursor charge set at 2+; and minimum peptides set at 2. Therefore, only two/more than two unique peptides were used to quantify proteins. The median intensities were set as normalization, and outliers were removed automatically. We considered any protein with more than 1.2-fold or less than 0.8-fold difference between 14 d/21 d and 7 d and having a *P*-value ≤ 0.05 to be differentially synthesized (Table S1).

### Bioinformatics analysis

We applied the Blast2GO program for functional annotations of the differentially synthesized proteins against a non-redundant database that comprised *Sus scrofa* proteins^35^. The energy pathway analysis was conducted using the KEGG Pathway database (http://www.genome.jp/kegg/). Biological process ontology of the differentially synthesized proteins was performed with the WEGO program, and Pearson Chi-Square tests were run to compare Terms between Down and Up groups^16^. Differences between values were considered statistically significant at *P* ≤ 0.05. Clustering of differentially synthesized proteins was conducted by Cluster 3.0 software, using k-means clustering (http://bonsai.hgc.jp/~mdehoon/sofware/cluster/sofware.htm).

## Electronic supplementary material

Supplementary Information

Dataset 1
