# Supplementary material for: Energy metabolism in the intestinal crypt epithelial cells of piglets during the suckling period
Source: Sci Rep. 2018 Aug 28;8:12948. doi: 10.1038/s41598-018-31068-3 (PMC6113243; doi:10.1038/s41598-018-31068-3)

**Supplementary Information**

**Energy metabolism in intestinal crypt epithelial cells of piglets during the suckling period**

**Qiye Wang^1^, Xia Xiong^2^, Jianzhong Li^1^, Qiang Tu^3^, Huansheng Yang^1,2^, Y. L. Yin^1,2^**

*^1^Hunan International Joint Laboratory of Animal Intestinal Ecology and Health, Animal Nutrition and Human Health Laboratory, School of Life Sciences, Hunan Normal University, Changsha, Hunan 410007, China;*

*^2^Key Laboratory of Agro-ecological Processes in Subtropical Region, Hunan Provincial Engineering Research Center of Healthy Livestock, Scientific Observing and Experimental Station of Animal Nutrition and Feed Science in South-Central, Ministry of Agriculture, Institute of Subtropical Agriculture, Chinese Academy of Sciences, Changsha, Hunan 410125, China*

**Correspondence: Yulong Yin*

*Chinese Academy of Science, Institute of Subtropical Agriculture*

*Yuandaer Road #644, 410125, Changsha, China*

*Tel: (86)-731-84619706*

*Fax: (86)-731-84612685*

*E-mail: yinyulong@isa.ac.cn (Y. Yin)*

**Figure S1.** Functional categorization of proteins in jejunal crypt epithelial cells of piglets during lactation. Differentially expressed proteins were grouped using Cluster 3.0 with k-means clustering. Any protein with ≥1.2-fold or ≤0.8-fold difference between 14 d or 21 d level and 7 d level (*P* ≤0.05) was considered differentially expressed.


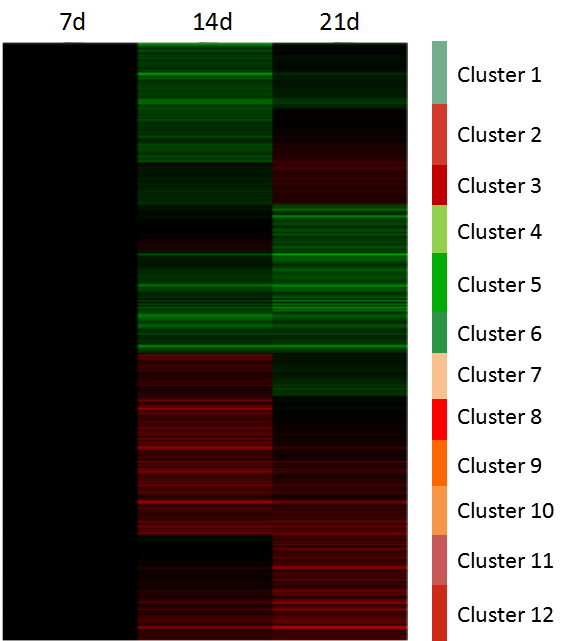

Supplement: Supplementary file 1 — Supplementary Information [file 41598_2018_31068_MOESM1_ESM.doc]
